# Enhanced ion acceleration from transparency-driven foils demonstrated at two ultraintense laser facilities

**DOI:** 10.1038/s41377-023-01083-9

**Published:** 2023-03-13

**Authors:** Nicholas P. Dover, Tim Ziegler, Stefan Assenbaum, Constantin Bernert, Stefan Bock, Florian-Emanuel Brack, Thomas E. Cowan, Emma J. Ditter, Marco Garten, Lennart Gaus, Ilja Goethel, George S. Hicks, Hiromitsu Kiriyama, Thomas Kluge, James K. Koga, Akira Kon, Kotaro Kondo, Stephan Kraft, Florian Kroll, Hazel F. Lowe, Josefine Metzkes-Ng, Tatsuhiko Miyatake, Zulfikar Najmudin, Thomas Püschel, Martin Rehwald, Marvin Reimold, Hironao Sakaki, Hans-Peter Schlenvoigt, Keiichiro Shiokawa, Marvin E. P. Umlandt, Ulrich Schramm, Karl Zeil, Mamiko Nishiuchi

**Affiliations:** 1 Kansai Photon Science Institute, National Institutes for Quantum Science and Technology, 8-1-7 Umemidai, Kizugawa, Kyoto 619-0215 Japan; 2 The John Adams Institute for Accelerator Science, Blackett Laboratory, Imperial College London, London, SW7 2AZ United Kingdom; 3 Helmholtz-Zentrum Dresden-Rossendorf, 01328 Dresden, Germany; 4 Technische Universität Dresden, 01069 Dresden, Germany; 5 Interdisciplinary Graduate School of Engineering Sciences, Kyushu University, 6-1, Kasuga-Koen, Kasuga, Fukuoka, 816-8580 Japan

**Keywords:** Plasma-based accelerators, Laser-produced plasmas, High-field lasers

## Abstract

Laser-driven ion sources are a rapidly developing technology producing high energy, high peak current beams. Their suitability for applications, such as compact medical accelerators, motivates development of robust acceleration schemes using widely available repetitive ultraintense femtosecond lasers. These applications not only require high beam energy, but also place demanding requirements on the source stability and controllability. This can be seriously affected by the laser temporal contrast, precluding the replication of ion acceleration performance on independent laser systems with otherwise similar parameters. Here, we present the experimental generation of *>*60 MeV protons and *>*30 MeV u^−1^ carbon ions from sub-micrometre thickness Formvar foils irradiated with laser intensities *>*10^21^ Wcm^2^. Ions are accelerated by an extreme localised space charge field ≳30 TVm^−1^, over a million times higher than used in conventional accelerators. The field is formed by a rapid expulsion of electrons from the target bulk due to relativistically induced transparency, in which relativistic corrections to the refractive index enables laser transmission through normally opaque plasma. We replicate the mechanism on two different laser facilities and show that the optimum target thickness decreases with improved laser contrast due to reduced pre-expansion. Our demonstration that energetic ions can be accelerated by this mechanism at different contrast levels relaxes laser requirements and indicates interaction parameters for realising application-specific beam delivery.

## Abstract (web summary)

High energy ions were accelerated by extreme space charge fields induced during relativistic transparency and optimised by tuning the target thickness to the individual laser parameters

## Graphical abstract

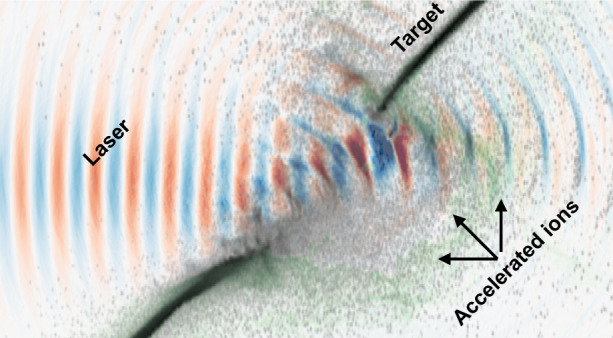

The advent of reliable high-quality high-power lasers has enabled rapid technological progress in their application to particle acceleration^1^. The development of ultrashort high peak current laser-driven ion sources has benefited from improvement in the delivery of ultraintense laser pulses combined with conceptual breakthroughs in controlling the interaction of extreme electromagnetic fields with matter^2,3^. The unique properties of laser-accelerated ion beams motivates numerous applications, including radiography of high energy density physics experiments^4^, ultrafast material response studies^5^, material processing^6^, injectors for accelerators^7,8^ and high dose rate radiobiology^9^.

A robust technique for generating energetic ions by laser irradiation is Target Normal Sheath Acceleration (TNSA)^10,11^. Electrons absorb energy from the laser at the front surface of a thin foil, and stream away from focus. As they exit the target, the charge imbalance drives a quasi-static electric field that accelerates surface ions, with maximum proton energies exceeding 70 MeV^12,13^. However, the mechanism’s modest intensity scaling^14–17^ makes it challenging to further increase the beam energy. Advanced acceleration schemes promise higher ion energies when using ultraintense pulses. For example, in light sail radiation pressure acceleration (LS-RPA) an opaque ultrathin target (thickness *d* << 100 nm) is collectively propelled by the laser^18,19^. However, such thin foils are extremely fragile and require an ultrahigh laser temporal contrast, the ratio of intensities between the main pulse and the preceding light. Furthermore, experiments and simulations with realistic laser parameters demonstrate difficulty in achieving coherent acceleration over the full duration of the laser pulse. Instead, target instabilities and heating of target electrons can cause rapid expansion, reducing the plasma core density and suppressing LS-RPA^20,21^. Eventually the electron density *n*_*e*_ drops below the relativistically corrected critical density *n*_cr_ = *γn*_c_, where *γ* is the electron Lorentz factor and *n*_c_ is the classical critical density, resulting in laser propagation through the target in the relativistically induced transparency (RIT) regime.

However, it has been shown that for targets driven in the RIT regime other acceleration mechanisms can also generate high ion energies, including volumetrically-enhanced sheath acceleration^22–24^, break-out afterburner (BOA)^25,26^, magnetic vortex acceleration (MVA)^27^, acceleration synchronised to the relativistic transparency front^28,29^, and hybrid mechanisms^30–32^. Experiments in the RIT regime have demonstrated carbon ions exceeding 1 GeV^33^ and proton beams approaching 100 MeV using high-energy picosecond-class laser systems^32^. Experiments with relatively low energy high-intensity femtosecond-class lasers have also demonstrated an impact of RIT on ion generation^34^, and recent simulation studies infer the importance of RIT over a wide range of laser intensities^35^.

Many applications of laser-driven ion sources require a transition from single-shot proof-of-principle studies to sustainable repetitive operation. The requirement of high repetition rate (*>*Hz) currently necessitates the use of femtosecond-class high-power laser drivers, which can provide high intensities but modest laser energies compared to typical glass-based picosecond-class systems. Despite recent advances in improving the inherent contrast of these laser systems^36,37^, they are not yet suitable for LS-RPA without applying single-use plasma mirrors^38^, complicating the setup and limiting the ultimate repetition rate. There is therefore great importance in optimising ion generation using femtosecond-class lasers, and in particular, investigating the RIT regime to maximise ion energies. Effective utilisation of this regime for applications also requires demonstration of sufficient control of the acceleration mechanism and understanding of the critical experimental parameters to enable replication at different facilities.

We report on the experimental generation of energetic protons exceeding 60 MeV and fully ionised carbon ions exceeding 30 MeV u^−1^ from relativistically transparent foils, reproduced on two state-of-the-art ultraintense femtosecond-class laser systems. We demonstrate that a careful choice of foil thickness matched to the contrast of the laser allows preparation of the target density for relativistic transparency, avoiding the need for plasma mirror systems. We verify the experimental results by hydrodynamic and 3D particle-in-cell (PIC) simulations showing that the most energetic particles are accelerated by the extreme space charge-induced electric field generated due to electron expulsion when the foil becomes transparent, followed by further acceleration in a diffuse sheath. We have replicated this regime on two different laser systems, highlighting the robustness of the mechanism. By actively controlling the laser contrast, we demonstrate a reduction in optimum target thickness when reducing the amount of laser prepulse. These results pave the way for the development of high-repetition laser-driven ion sources exploiting the relativistically induced transparency regime.

## Results

### Target thickness-dependent ion acceleration

**Fig. 1:**
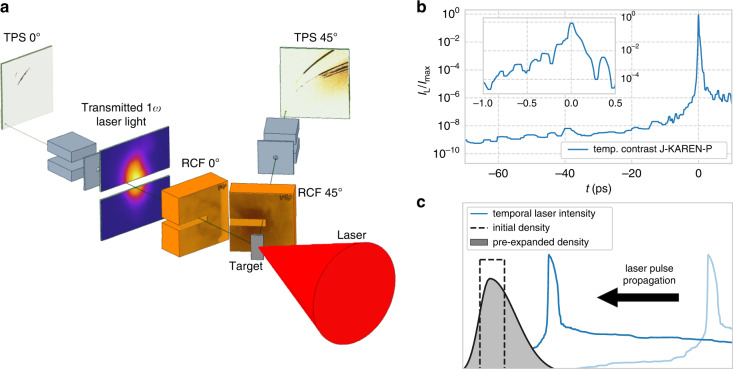
Sketch of the experiment. **a** The J-KAREN-P laser was focused onto Formvar films at 45°. Accelerated particles were detected spatially by two radiochromic films (RCF) stacks and spectrally by two Thomson Parabola Spectrometers (TPS) oriented at 0° and 45° with respect to the laser axis. When RCF is not used, a ground glass screen diffusely scattered transmitted laser light for detection by a filtered CCD camera. **b** Temporal intensity contrast of J-KAREN-P on the sub 70 ps and sub 1 ps scale (inset) measured by third-order auto-correlation and spectral interferometry, respectively. **c** Illustration of prepulse-induced expansion of the target before main pulse arrival

The effect of changing target thickness on ion acceleration was investigated using the J-KAREN-P laser at the Kansai Photon Science Institute^39^. The experimental setup is shown in Fig. 1a (details in Methods). Laser pulses with 10 J on-target energy and 45 fs Full-Width-Half-Maximum (FWHM) duration were focused by an *f*/1.4 off-axis parabola onto Formvar targets (C_5_H_8_O_2_, density *ρ* ≈ 1.25 gcm^*−*3^) placed at 45° to the incident laser, enabling separation of observable acceleration mechanisms^30^. The focal spot diameter was ≈1.5 µm FWHM, resulting in a peak intensity of 3.5 × 10^21^ Wcm^−2^. The laser-plasma interaction and ion acceleration were measured by optical and particle diagnostics placed around the target.

The laser was used with inherent contrast, i.e. without a contrast-enhancing plasma mirror system. The temporal laser intensity on-target relative to the peak of the pulse is shown in Fig. 1b. The prepulse, defined here as light preceding the main pulse, ionises and heats the target. This results in significant target decompression before the arrival of the peak of the pulse (Fig. 1c). Therefore, changing the initial target thickness varies not only the areal density, but also the peak density of the target interacting with the main pulse.

**Fig. 2:**
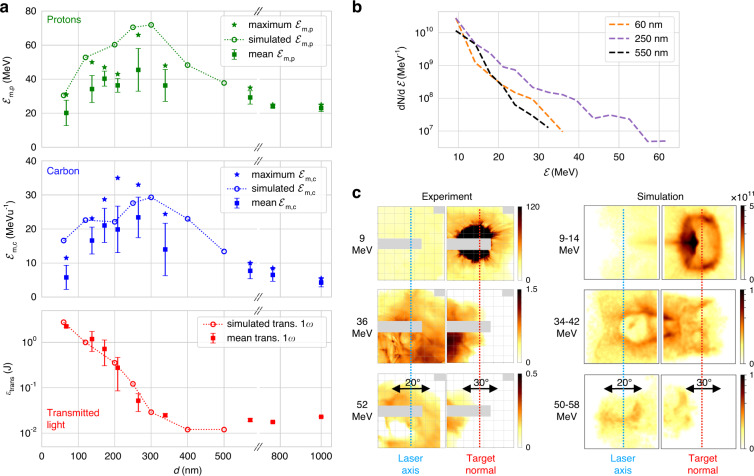
Experimental observation of accelerated ions. **a** Maximum ion energies per nucleon ℰ_m_ recorded on the TPS detectors for different target thicknesses, which for *d* < 700 nm was on the 0° TPS and for *d* > 700 nm on the 45° TPS, for protons (top) and carbon (middle), together with the corresponding transmitted 1*ω* laser energy *Ɛ*_trans_ (bottom). Error bars represent the standard deviation and stars show maximum ℰ_m_ of shots in each thickness bin. Over 50 shots are included over the entire thickness range. Circles show the corresponding ℰ_m_ and *Ɛ*_trans_ from 3D PIC simulations. **b** Proton spectra from RCF, combining the signal on both RCF stacks, for three example target thicknesses. **c** Proton beam spatial profile at different energies for *d* = 250 nm. The experimental data (left, same shot as purple line in **b**) gives dose (Gy) from RCF at different Bragg peak energies. The grey shaded area indicates regions not measured by the RCF. Equivalent synthesised profiles from 3D PIC (right) give the number of particles per steradian over the indicated energy range. The ring feature from 9 to 14 MeV is due to the small simulation box size, which artificially limits the transverse extent of the sheath at the rear surface

The maximum detected energies per nucleon ℰ_m_ of the two dominant particle species (protons and *Z/A* = 0.5 ions dominated by C^6+^) and the energy of 1*ω* transmitted laser light (*Ɛ*_trans_) as a function of initial target thickness *d* are plotted in Fig. 2a. There is an optimal thickness *d*_opt_ ≈ 250 nm where the energies of both species are maximised, reaching ℰ_m,p_ > 60 MeV for protons and ℰ_m,c_ > 30 MeV u^−1^ for carbons. Thinner or thicker targets result in lower energies for both species. Whereas for *d* < 700 nm the most energetic particles were found on the 0° TPS, for *d* > 700 nm particles were only observed on the 45° TPS. The amount of transmitted light also indicates a clear thickness dependency. While targets with *d* > 300 nm show a constant and nearly negligible amount of transmitted light (*Ɛ*_trans_ *<* 30 mJ ≃0.3% input energy), transmission increases exponentially for thinner targets and reaches *Ɛ*_trans_ >> 500 mJ (*>*5% of input energy) for *d* < 200 nm. This sudden increase in transmission for target thicknesses between 200 nm and 300 nm indicates a pronounced decrease in the peak density of the targets at the arrival of the main pulse. There is a clear correlation between the onset of transparency and a dramatic increase in the generated beam energies. However, for very thin targets with high transmission, the ion energy reduces again. The measurable but relatively low transmission for optimum ion energies indicates an efficient conversion of laser energy in the transparent but still dense target.

Full-beam energy spectra deconvolved from RCF stacks for three representative shots at different target thicknesses are shown in Fig. 2b. Targets that are highly transmissive (*d* = 60 nm) or opaque (*d* = 550 nm) show a similar spectral shape and ℰ_m,p_ ≈ 35 MeV. In contrast, for *d* = *d*_opt_, there is an increase in both cutoff energy, to ℰ_m,p_ ≈ 60 MeV, as well as particle flux at high energies, while maintaining a thermal spectral shape. An example of the energy-dependent spatial-dose distribution of protons generated at the optimum target thickness (*d* = 250 nm) is shown in Fig. 2c, together with a synthetic spatial beam profile derived from PIC simulations (discussed below). Lower energy protons (9 MeV) show a confined round beam profile along the target normal direction with a low flux divergent emission evident along the laser axis. With increasing particle energy, the beam profile centre shifts towards the laser axis, with significant spatial structure apparent. At the highest energies (e.g. 52 MeV), the beam is directed primarily along the laser axis. This observation of the most energetic protons propagating closer to the laser axis is consistent with TPS data, which shows significantly higher energies along laser axis than target normal for targets *d* ≈ *d*_opt_ (Supplementary Fig. S1). Conversely, for the thickest targets where laser transmission is negligible, no protons are observed along the laser axis. The TPS also confirms that the beam direction dependence on-target thickness is the same for accelerated carbon ions. This change in beam directionality and shape, correlated with the increase in transmitted light, suggests a strong influence on the acceleration mechanism due to relativistically induced transparency.

### Elucidating target pre-expansion and ion acceleration in the relativistically induced transparency regime

Understanding the pre-expansion of the target is essential for elucidating the high-intensity laser-plasma interaction at the peak of the pulse. When incident on a transparent dielectric target, laser prepulse will cause ionisation before the peak of the pulse hits the target via laser induced breakdown (LIB)^40^. The timing of LIB was derived from a comprehensive pre-study to be ~67 ps before the main pulse arrival (see Methods). The prepulse prior to this time (see Supplemental Fig. S2) does not significantly affect the foil. After LIB, free electrons absorb laser energy on the target front surface, leading to ablation and the generation of a shockwave propagating into the target. For sufficiently thin targets, the shock can breakthrough the back of the target, causing a reduction of the core density before the arrival of the peak of the pulse. We implemented a two-stage simulation method, performing hydrodynamic simulations of the low-intensity rising edge after LIB (Fig. 1b) followed by 3D particle-in-cell (PIC) simulations of the final high-intensity ramp and main pulse using the FLASH and EPOCH codes respectively. FLASH is used in 2D cylindrical geometry and models the prepulse absorption using ray tracing coupled to an inverse bremsstrahlung heating model. A tabulated Formvar EOS was generated using the FEOS code^41^. The resultant density profile at 1 ps before the main pulse was then used to initialise the 3D PIC simulations of the high-intensity interaction. Further details of the simulation methodology are provided in the Methods.

**Fig. 3:**
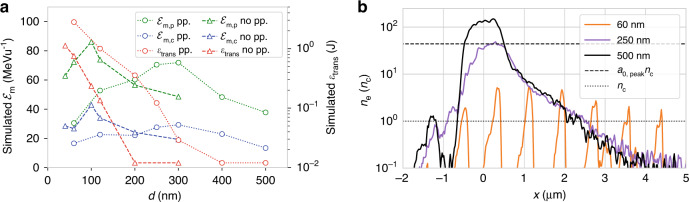
Modelling target pre-expansion. **a** Comparison of simulations including laser prepulse (dotted/circles) and simulations without prepulse starting 50 fs before the peak of the pulse (dashed/triangles), showing maximum carbon and proton energies ℰ_m_ and transmitted laser energy *Ɛ*_trans_ as a function of target thickness *d*. **b** Electron density lineouts 35 fs before the arrival of the peak of pulse for different target thicknesses. For *d* = 60 nm, the target is already transparent, resulting in electron bunching

The variation in ℰ_m_ and *Ɛ*_trans_ with changing *d* from the two-stage simulations is shown overlaid (circles) on the experimental data in Fig. 2a. There is good agreement with the experiment over the entire thickness range. The simulations accurately recreate the optimum target thickness and maximum ion energies. The plotted simulation data gives the maximum energy in any angular direction, whereas the TPS measurements are restricted to 0° and 45°. For simulations of targets with *d* > *d*_opt_ the highest energies were often found between laser axis and target normal. The effect of not including the prepulse in the simulation is shown in Fig. 3a. Without prepulse, there is a significant reduction in optimum thickness to ≈100 nm, as well as a slight increase in ℰ_m_. Evidently, including prepulse-driven target expansion is essential to accurately model the experiment.

To illustrate the impact of the prepulse on the density distribution, Fig. 3b shows the simulated electron density from a lineout through the central axis of the target 35 fs before laser peak arrival for three initial target thicknesses. For the thickest target, *d* = 500 nm, although displaying a reduction in peak density and formation of significant scale lengths on the front and rear surface, the core density still greatly exceeds the roughly estimated relativistic critical density of the peak of the pulse *n*_cr_ ≈ *a*_0,peak_*n*_c_, where *a*_0,peak_ is the normalised vector potential at the peak of the pulse and assuming an electron *γ* ≈ *a*_0_ for *a*_0_ >> 1. The ensuing intense laser-plasma interaction is therefore with a fully opaque plasma. For *d* = 250 nm, although still opaque at this stage, the peak density is very well matched with the estimated *n*_cr_ at the peak of the pulse, implying a relativistically transparent interaction. However, for the thinnest target *d* = 60 nm, bunching at the laser frequency is observed even before the peak of the pulse. The target centre is already transparent, with time-averaged densities lower than the classical critical density. This demonstrates that a combination of adjusting the target thickness and utilising the inherent contrast provides a method to prime plasma for relativistic transparency. Detailed characterization and treatment of the prepulse are therefore essential to predict the target density distribution at the arrival of the main pulse.

**Fig. 4:**
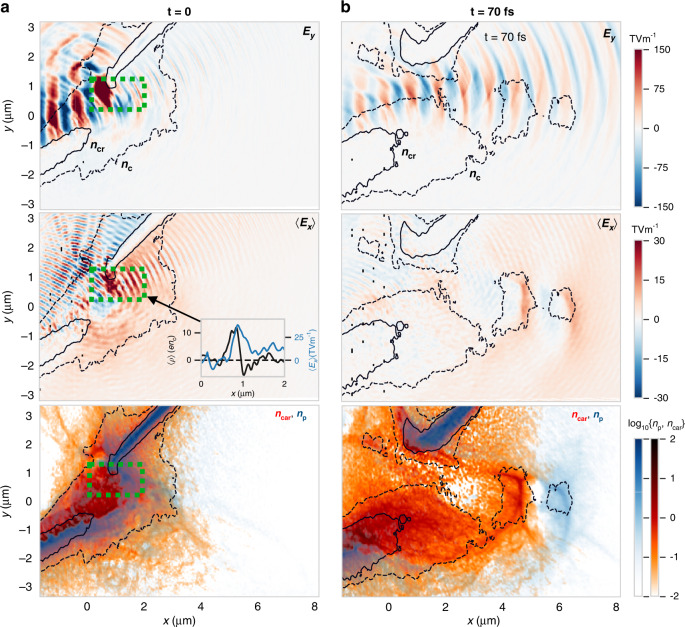
Dynamics of laser-plasma interaction at optimum target thickness. **a** 2D slice (*z* = 0) of *E*_*y*_, 〈*E*_*x*_〉 and carbon/proton density (*n*_car_, *n*_p_ normalised to *n*_c_) for a 250 nm target at *t* = 0, when the peak of the pulse arrives. The solid (dashed) contours give the relativistic (classical) critical electron density. The region of strong electron expulsion is highlighted by the green box. The inset shows the cycle-averaged space charge density 〈*ρ*〉 and 〈*E*_*x*_〉 for a lineout through the centre of the green box, averaging over ∆*y* = 400 nm. **b** The same 2D slices shown at *t* = 70 fs, at the end of the main pulse interaction, showing the acceleration of the separated ion species in the thermal sheath fields

To identify the ion acceleration process for the optimum thickness, we now describe the main pulse interaction with the pre-expanded plasma for *d* = *d*_opt_ = 250 nm. During the femtosecond rising edge before the peak of the pulse, the laser is strongly absorbed by a still opaque plasma. The ultrahigh intensities result in a strong *v* × *B* radiation pressure, bunching and accelerating the electrons along the laser axis. Energetic electrons generate sheath fields, accelerating ions and causing a rapid expansion of the target. A 2D slice from the simulation at the peak of the pulse is shown in Fig. 4a. As shown by the electron density contours, the electrons in the centre of the target have been heated and expanded such that *n*_c_ < *n*_e_ < *n*_cr_, and the laser has started to penetrate the target. The laser volumetrically interacts with target electrons, leading to strong absorption. This results in the *v* × *B* driven electrons being rapidly expelled from the focal region. As the ions are not significantly accelerated by the laser fields due to their lower charge-to-mass ratio, a region of large space charge develops in the transparent region highlighted by the green box. This space charge is maintained by the continuous expulsion of electrons due to the radiation pressure. Although the expulsion of electrons is not complete, unlike total expulsion in the Coulomb Explosion regime^42,43^, the magnitude of the time-averaged space charge can reach 〈*ρ*〉 *>*10*en*_c_, which results in a region of large cycle-averaged quasi-static electric field 〈*E*_*x*_〉 ~30 TV m^−1^, with a spatial extent of ~500 × 500 × 500 nm^3^ (see inset in Fig. 4a). The ions also experience an oscillating longitudinal field *E*_*x*_ as the forward moving relativistic electron bunches pass through them, without gaining net energy. Due to their higher charge-to-mass ratio, the protons have started to separate from the more abundant carbon ions, but they are still both present in the region of strong 〈*E*_*x*_〉. Additionally, there is a weaker 〈*E*_*x*_〉 apparent over a large extent at the target rear. These are characteristic pre-thermal sheath fields^44^ along the long density scale lengths at the rear surface sustained by electron pressure, differentiating it from the stronger localised field due to radiation pressure sustained electron expulsion.

After the end of the most intense part of the laser pulse, acceleration continues in the now thermally driven sheath, with significantly lower electric fields. Complete species separation becomes prominent due to the faster velocity of the protons, as shown in Fig. 4b. At this point, the bunching of the forward accelerated protons and ions results in two distinct sheath fields around each species, with 〈*E*_*x*_〉 *<*10 TV m^−1^.

**Fig. 5:**
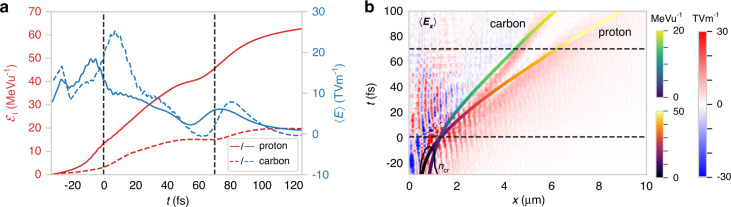
Acceleration of most energetic particles for optimum target thickness. **a** Time history of kinetic energy ℰ_i_ (red) and local electric field 〈*E*〉 (blue) experienced by exemplary proton (solid lines) and carbon ion (dashed). **b** Time history of 〈*E*_*x*_〉 along *x* lineout through the region of largest accelerating gradient, with overlaid trajectory of the same proton and carbon ion. The solid black line shows the *n*_cr_ contour. Black dashed lines indicate the time of the two snapshots in Fig. 4

Particle tracking reveals that all the most energetic carbon ions and protons originate from the region of strong electric field generated by electron expulsion in the ion core during relativistic transparency. The time history of the kinetic energy of exemplary proton and carbon ions is given in Fig. 5a, along with the cycle-averaged electric field driving the acceleration. The ions are accelerated by strong fields up to 〈*E*〉 ~20 TV m^−1^ around the peak of the pulse. These are the fields sustained by the electron expulsion, and they provide a significant kick to the ions. The relative time offset between the peak of the field experienced by the proton and carbon (blue lines) is due to an offset in starting position of the selected particles, as the laser-sustained space charge moves along with the expanding ions. Figure 5b shows a waterfall plot of 〈*E*_*x*_〉 along a line through the centre of acceleration (*z* = 0, *y* = 0.7 µm) as a function of time, overlaid with the *n*_cr_ contour. The strongest field is generated near the peak of the pulse (*t* = 0), just after transparency occurs. As the target ions are accelerated forwards, the laser-sustained space charge also moves forwards, decreasing as the ion density decreases. The ions in this region are therefore in a moving potential, increasing their energy gain. Eventually, at *t* ≈ 20 fs, both the ion density and the laser pressure have decreased to such an extent that a strong localised field is no longer apparent above the thermally driven sheath.

After this point, the ions continue to be accelerated by the ambient sheath fields. The ions which received the largest velocity kick from the laser-sustained space charge record a further energy gain as they are accelerated through weaker sheath fields *E* < 10 TV m^−1^, as seen in Fig. 5b. Therefore, at *d* ≈ *d*_opt_, the majority of the most energetic ions are accelerated by a hybrid mechanism—a swift acceleration due to laser-driven electron expulsion in the dense transparent plasma core followed by further acceleration in the diffuse sheath.

Proton beam spatial profiles synthetically generated from the simulated particle distribution, assuming ballistic motion after the end of the simulation, are shown in Fig. 2c. Similar to the experiment, the lower energies show a dominant emission towards the target normal direction and are due to protons accelerated by the diffuse sheath at the periphery of the target at radii larger than the laser focal spot size. This beam is limited to energies *<*20 MeV. For beam profiles at higher energies, the target normal beam is not seen. Instead, a divergent and diffuse beam is observed directed predominantly along the laser axis.

For targets thicker than the optimum, for example, *d* = 500 nm, the simulations show that the plasma remains opaque throughout the interaction. There is high laser absorption into electron kinetic energy, and this drives large diffuse thermally driven sheath fields on the rear side of the target. No significant transmitted laser light is observed, and there is no contribution of relativistic transparency to the ion acceleration. On the other hand, when targets are too thin, for example, *d* = 60 nm, the target is already well below the classical critical density by the peak of the pulse. There is still significant absorption in the target, as electrons are injected from the periphery of the relativistic plasma aperture into the strong laser fields^45^. The accelerated electrons again contribute strongly to widespread thermally driven diffuse sheath fields, but the ion density in the target remnants is too small to generate the larger amplitude localised space charge field.

### Experimental demonstration of prepulse dependence on optimal target thickness

We have therefore shown that the key to maximising ion energies is the pre-expansion of the targets to match the relativistically corrected critical density at the peak of the pulse. Therefore, *d*_opt_ is expected to be dependent on both the laser contrast, which controls the maximum target density, and the laser intensity, which controls the electron heating and therefore the density required for transparency. We investigated this using a different laser capable of varying the temporal laser contrast. The DRACO-PW laser at the Helmholtz-Zentrum Dresden-Rossendorf ^13,46^ has comparable laser parameters to the J-KAREN-P laser (see Methods) and is therefore ideally suited for a complementary study. In particular, the prepulse contrast level on the multi-ps timescale is very similar on both systems (see Supplemental Fig. S3), implying the same pre-expansion should occur. The laser intensity is also slightly higher on DRACO-PW (*I*_*L*_ ≈ 5 × 10^21^ W cm^−2^). Additionally, there is an option for on-demand contrast improvement using a single-shot plasma mirror system, reducing the prepulse level by almost four orders of magnitude and therefore suppressing target pre-expansion. This enabled two different preplasma conditions to be investigated. The setup at DRACO-PW used an identical target irradiation geometry as the J-KAREN-P experiment and similar diagnostics, facilitating comparable measurements.

**Fig. 6:**
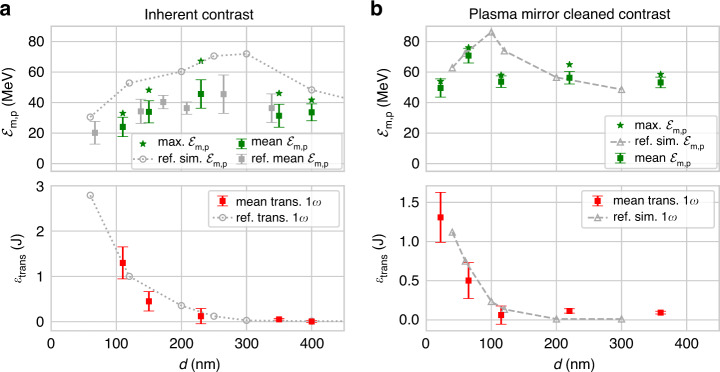
DRACO-PW experiments studying pre-expansion influence. Measurements of maximum proton energy ℰ_m,p_ (green squares) and transmitted 1*ω* light *Ɛ*_trans_ *(*red squares) for different target thicknesses *d* from experiments at DRACO-PW for **a** inherent and **b** plasma mirror cleaned temporal contrast. Reference J-KAREN-P proton energy data (grey squares) is included for comparison. The error bars represent the standard deviation of mean values (squares), stars show maximum values of ℰ_m,p_. Grey circles and triangles show reference ℰ_m,p_ and *Ɛ*_trans_ from the 3D PIC simulations presented in Fig. 3a

Firstly, we show the results using a matched prepulse level to J-KAREN-P. The maximum proton energies and the amount of transmitted 1*ω* light dependency on-target thickness *d* is depicted in Fig. 6a.

A pronounced optimum thickness *d*_opt_ ≈ 230 nm is observed. The target thickness dependency closely matches the J-KAREN-P experiment and simulation (overlaid in grey). The proton energies are comparable to the J-KAREN-P experiment, albeit with a slightly higher maximum energy ℰ_m_ ≈ 70 MeV. The thickness dependency of *Ɛ*_trans_ is also similar to previous observations. While thicker targets have a low and almost unvarying transmitted light signal, there is a rapid increase in transmission at *d* ≈ *d*_opt_. For decreasing thickness, *Ɛ*_trans_ continues to increase while ℰ_m,p_ decreases. The agreement of these key parameters indicates that the target was in a pre-expanded state as described for the J-KAREN-P experiments. The striking similarity of the results from two different laser facilities with similar temporal contrast implies robustness of the acceleration scheme.

We then investigated the influence of an improved temporal contrast by introducing the plasma mirror system. Figure 6b shows the effect of improved contrast on ℰ_m,p_ and *Ɛ*_trans_ with respect to *d*. Between 360 nm and 115 nm, a plateau-like region can be identified where no transmission occurs and ℰ_m_ ≈ 55 MeV. Similar to before, a region of optimal acceleration performance exists, where the proton energies show a peak and the amount of transmitted light starts to increase. Compared to experiments with intrinsic laser contrast, the optimum thickness is reduced to *d*_opt_ ≈ 65 nm for which ℰ_m,p_ = 76 MeV. Targets with *d* < *d*_opt_ show significant transmitted light (up to 1.6 J ≃ 9% of input energy) and a reduction in ℰ_m,p_. These results show a high level of agreement with the simulations neglecting prepulse (grey triangles). This demonstrates that the plasma mirror-induced contrast cleaning limited the plasma dynamics to the last ps before the main pulse. A detailed analysis of the simulations neglecting the prepulse (see Supplemental Fig. S4 and S5) reveal similar plasma and field dynamics to the optimised pre-expanded target, but for a lower *d*_opt_, for which the lower areal mass resulted in relativistic transparency at the peak of the pulse due to target expansion in the femtosecond rising edge.

## Discussion

Our results demonstrate that having relativistic transparency occur at the peak of the pulse is strikingly beneficial. This is similar to what has been observed for lower-intensity picosecond-class lasers where volumetric laser heating^22^ or hybrid RPA-TNSA^32^ results in higher ion energies. Here, however, during the ultraintense femtosecond pulse interaction, the largest accelerating gradients and energy gain dominantly arises from significantly higher space charge fields resulting from electron expulsion from the relativistically transparent target core, albeit with a smaller contribution from the diffuse sheath fields at later times. Unlike the BOA regime^26,31^, there are no apparent low-frequency electrostatic wave structures resonant with the accelerated ions. However, the enhanced ion generation from all these mechanisms share similar experimental phenomenology, including an optimal ion energy via timing the transparency time to the peak of the pulse, and measurable laser transmission through the target.

The RIT-enhanced acceleration mechanism described here is a promising regime for application-oriented high energy ion acceleration. In contrast to recent efforts to use plasma mirrors to minimise the prepulse of high power femtosecond lasers to enable irradiation of few nanometre thick targets, we show that these lasers can unexpectedly be used in a different acceleration regime with relaxed contrast requirements. This not only significantly reduces the complexity, size and cost of the ion source but also removes one of the limits to continuous high repetition rate operation. The required target thickness, a few hundred nanometres, improves robustness compared to the *d* ~10 nm targets required for LS-RPA at the same intensity range, which are easily damaged during handling, pump down or by preceding shots on the target system.

The successful replication at two different laser facilities demonstrates that this acceleration regime is suitable for existing high-power femtosecond-class lasers. The similarity in the results from J-KAREN-P and DRACO-PW systems, despite different laser front-end configurations and slightly different focal spot parameters, reveals the robustness of the regime. Our investigation of the prepulse-induced density shaping and acceleration mechanism implies that laser contrast and intensity are the most important factors for determining the optimal target thickness for this regime. The laser contrast determines the target density at the peak of the pulse for a given target thickness, whereas the laser intensity determines the density required for relativistic transparency. Although the target pre-expansion is sensitive to laser contrast which varies between facilities, it is possible to choose a prepulse-adapted thickness to match target density to laser intensity and hence prime the target for relativistically induced transparency at laser peak arrival. The combination of acceleration mechanisms and the multi-variate scans required to optimise the target thickness for different laser conditions present challenges for measuring the ion energy scaling with laser parameters, which remains an important topic for future investigation.

In summary, we have investigated ion acceleration from the interaction of an ultraintense femtosecond laser pulse with thin Formvar foils and found an optimised target thickness for the acceleration of protons and carbon ions *>*60 MeV and *>*30 MeV u^−1^ respectively. We determined that the laser prepulse plays an essential role in priming the plasma density for relativistic transparency. Improved contrast reduced the optimal target thickness with only a minor improvement in the maximum proton energies. The acceleration of the highest energy ions is due to a strong space charge field generated by the expulsion of electrons during relativistically induced transparency, followed by further acceleration in an ambient sheath. Our reproduction of the acceleration regime on two different lasers underlines its robustness and demonstrates that plasma mirrors and ultrathin nanometre scale targets are not required for high energy ion acceleration using femtosecond-class laser systems. This conceptual breakthrough establishes a path towards the development of ~100 MeV class repetitive ion sources using currently available laser technology.

## Methods

### Laser system and pulse characterisation

Experiments were performed at two different institutes. The J-KAREN-P laser at the Kansai Photon Science Institute, QST, Japan is based on a hybrid OPCPA/Ti:Sa architecture (central wavelength 800 nm), and delivered pulses of 10 J energy on target and ≈(45 ± 5) fs FWHM pulse duration at 0.1 Hz repetition rate^39^. The pulses are focused by an *f*/1.4 off-axis parabola (OAP) to a spot size of ≈1.5 µm FWHM resulting in a peak intensity of (3.5 ± 0.5) × 10^21^ Wcm^−2 ^^47^.

Complementary experiments were performed at the DRACO-PW laser at the Helmholtz-Zentrum Dresden-Rossendorf in Dresden, Germany. DRACO-PW is a double CPA Ti:Sa laser system, providing 30 fs (FWHM) laser pulses with a central wavelength of 810 nm (50 nm FWHM bandwidth). On-demand plasma mirror cleaning can improve the temporal contrast by almost 4 orders of magnitude, essentially removing all remaining prepulses and limiting the ionisation and plasma dynamics to the last ps before the peak of the laser (intensity below 10^16^ W cm^−2^ at −1 ps). To compensate for laser energy loss in the PM, the input laser energy was increased to provide matched pulse energy and intensity at target. Laser pulses with an on-target energy of 17.6 J (inherent contrast) or 18 J (plasma mirror cleaned contrast) were focused by an *f*/2.3 OAP to a FWHM spot size of 2.5 µm (2.6 µm) yielding peak intensities of 5.1 × 10^21^ (5.4 × 10^21^) W cm^−2^.

The temporal contrast of the individual laser systems was characterised with identical measurement methods and instruments to ensure comparability. For this purpose, a 1-inch silver mirror was used to pick off a central part of the collimated laser beam before the final focusing optic and send it to various diagnostics. Third-order auto-correlator (TOAC) measurements (Sequoia and SequoiaHD from Amplitude Technologies) enabled prepulse identification with high dynamic range up to several 100 ps before the peak of the pulse via temporal scanning (for an attenuated beam), shown in Supplemental Fig. S2. The baseline temporal contrast intensity at *>*100 ps before the peak is below the detection limit of the detector. Supplemental Fig. S3 shows the temporal contrast comparison after laser induced breakdown in more detail. From −60 ps until −10 ps the intensity contrast is increasing from below 10^*−*9^ until 10^*−*8^ and to ≈10^*−*5^ at −1 ps. Spectral phase interferometry for direct electric-field reconstruction (SPIDER) and self-referenced spectral interferometry with extended time excursion (SRSI-ETE)^48^ measurements techniques provided information about the contrast level several ps and sub-ps before the main pulse on a single-shot basis.

### Targets and diagnostics

The intense laser pulses irradiated Formvar plastic foils (C_5_H_8_O_2_, *n*_e_ = 230 *n*_c_ when fully ionised) in a thickness range from *d* = 20 nm to 1000 nm at an incidence angle of 45° with p-polarisation. Two Thomson parabola spectrometers (TPS) positioned at 0° and 45° with respect to the laser axis measured the proton and ion energy spectra. Over the whole thickness range investigated, the prominent light ion species had *Z/A* = 0.5. Considering the target composition, this is dominated by C^6+^ with a contribution from O^8+^. In the text, these are labelled as carbon for simplicity. At J-KAREN-P, BAS-SR Fujifilm imaging plate was used as the detector, while at DRACO-PW a multi-channel plate detector was used.

The spatial proton intensity distribution was measured by two radiochromic film (RCF) stacks placed behind the target, one along laser axis and the other along target normal at a distance of 100 mm and 60 mm respectively. This enabled discretised energy detection in the full opening angle between laser forward and target normal direction. The XR-RV3 films were 70 × 70 mm in size, with a horizontal slot through the centre to enable simultaneous measurement with the TPS.

Fundamental laser light through the target was scattered off ground glass placed ≈50 cm behind the target (J-KAREN-P) or a ceramic screen placed ≈33 cm behind the target (DRACO-PW), and then imaged onto a bandpass filtered (800 ± 25 nm) and calibrated camera.

### Laser-induced breakdown time

To successfully perform a start-to-end simulation of high-intensity laser-solid interactions, it is essential to know the time at which the laser induced breakdown (LIB) occurs, when the initially solid-state material transitions into the plasma state. We used targets made of Formvar, a transparent dielectric with a high band gap, which is transmissive to incoming laser light until LIB occurs. The LIB threshold for different pulse durations was determined from experimental optical probing during a dedicated pre-study at DRACO-PW using 300 ± 50 nm Formvar foils. Comparing those values with the laser temporal contrast, shown in Supplemental Fig. S2, indicates that the threshold is reached between 70 ps and 65 ps. Although, a dedicated LIB study was not performed at J-KAREN-P, the similarity of the contrast to DRACO-PW implies a similar LIB time. We also note that there exists no femtosecond prepulses sufficient for LIB prior to 70 ps, and the *>*100 ps amplified spontaneous emission is significantly lower than the relevant LIB threshold^49^. Supplemental Fig. S3 shows the time period after LIB, highlighting the similarity of the subsequent contrast of the two laser systems. Therefore, the following prepulse-driven plasma expansion will proceed similarly in both experiments.

We note that different dielectrics or metal targets would be affected by the prepulse differently due to different breakdown times and material properties^49^. The importance of understanding this process in elucidating the high-intensity laser-plasma interaction motivates detailed material-dependent studies of the LIB process in comparison with high dynamic range, full-power measurements of the temporal contrast.

### Numerical simulations

The numerical modelling of the experiment was performed using two different codes. Initially, the FLASH code (v4.6.2)^50^ was used in a 2D radially symmetric geometry with adaptive mesh refinement to perform hydrodynamic simulations of the lower intensity prepulse from the onset of LIB at 67 ps up to 1 ps before the main pulse. The Lee-More conductivity and heat exchange were used. As is typical in such codes, the laser absorption was modelled by a ray tracing method, in which electrons are directly heated by inverse bremsstrahlung heating. Wave effects, such as resonance heating, are not included, which may slightly underestimate the heating absorption rate. However, a small uncertainty in absorption does not make a significant difference in simulations that vary laser intensity over many orders of magnitude.

The final picosecond and main pulse interaction were modelled using EPOCH 3D (v4.17)^51^. A simulation box of 30 × 25 × 14 µm was simulated with a spatial resolution of 20 × 40 × 40 nm, where the highest spatial resolution was along the laser propagation direction. The cylindrically symmetric density profile from the hydrosimulation was mapped into 3D and used as the initial density profile, assuming full ionisation. It was assumed that there was no species separation, and electrons, carbon/oxygen ions and protons were initialised with 14, 8, and 8 particles per cell respectively. To minimise the computational time, regions of the density profile with fully ionised density less than 1.0 *n*_c_ were removed during initialisation. The laser was focused to a Gaussian spot (1.5 µm FWHM) on the front surface of the target in p-polarisation, with a peak intensity *I*_L_ = 4 × 10^21^ Wcm^−2^. The intensity temporal profile was matched to an average single-shot measurement from the SRSI-ETE diagnostic, shown inset in Fig. 1a.

It is important to note that due to the wide range of laser intensities and target conditions present during the prepulse interaction, current widely used techniques for modelling the prepulse have various limitations. For example, hydrodynamic codes often use oversimplified models for laser heating and behaviour of the low-temperature solid bulk before target decompression. The rising edge always goes through a range of intensities between ≈10^15^ to 10^17^ Wcm^−2^ where the target is still relatively cold but nonlocal heating can be important, a regime extremely difficult to model with either hydrodynamics or PIC calculations. Therefore, further development of both experimental and numerical methods could significantly improve understanding of the target condition at the peak of the pulse and enable increased understanding of source stability and optimisation.

## Supplementary information

Supplemental figures

## Data Availability

The experimental and simulation data that support the findings of this study are available from the corresponding author upon reasonable request.
