# Supplementary material for: Enhanced ion acceleration from transparency-driven foils demonstrated at two ultraintense laser facilities
Source: Light Sci Appl. 2023 Mar 13;12:71. doi: 10.1038/s41377-023-01083-9 (PMC10011581; doi:10.1038/s41377-023-01083-9)
Supplement: Supplementary file 1 — Supplemental figures [file 41377_2023_1083_MOESM1_ESM.pdf]

Supplementary Information for **Enhanced ion acceleration from transparency-driven foils demonstrated at two ultraintense laser facilities**

Nicholas P. Dover[1,2,*], Tim Ziegler[3,4,*], Stefan Assenbaum[3,4], Constantin Bernert[3,4], Stefan Bock[3], Florian-Emanuel Brack[3,4], Thomas E. Cowan[3,4], Emma J. Ditter[2], Marco Garten[3,4], Lennart Gaus[3,4], Ilja Goethel[3,4], George S. Hicks[2], Hiromitsu Kiriyama[1], Thomas Kluge[3], James K. Koga[1], Akira Kon[1], Kotaro Kondo[1], Stephan Kraft[3], Florian Kroll[3], Hazel F. Lowe[1], Josefine Metzkes-Ng[3], Tatsuhiko Miyatake[1,5], Zulfikar Najmudin[2], Thomas Püschel[3], Martin Rehwald[3,4], Marvin Reimold[3,4], Hironao Sakaki[1,5], Hans-Peter Schlenvoigt[3], Keiichiro Shiokawa[1,5], Marvin E. P. Umlandt[3,4], Ulrich Schramm[3,4], Karl Zeil[3,†], and Mamiko Nishiuchi[1,†]

[1]Kansai Photon Science Institute, National Institutes for Quantum Science and Technology, 8-1-7 Umemidai, Kizugawa, Kyoto 619-0215, Japan
[2]The John Adams Institute for Accelerator Science, Blackett Laboratory, Imperial College London, London SW7 2AZ, United Kingdom
[3]Helmholtz-Zentrum Dresden-Rossendorf, 01328 Dresden, Germany
[4]Technische Universität Dresden, 01069 Dresden, Germany
[5]Interdisciplinary Graduate School of Engineering Sciences, Kyushu University, 6-1, Kasuga-Koen, Kasuga, Fukuoka 816-8580, Japan

[*]These authors contributed equally to this work
[†] k.zeil@hzdr.de; nishiuchi.mamiko@qst.go.jp

Supplementary Figure 1: Comparison of ion acceleration at 0° and 45° from J-KAREN-P experiment

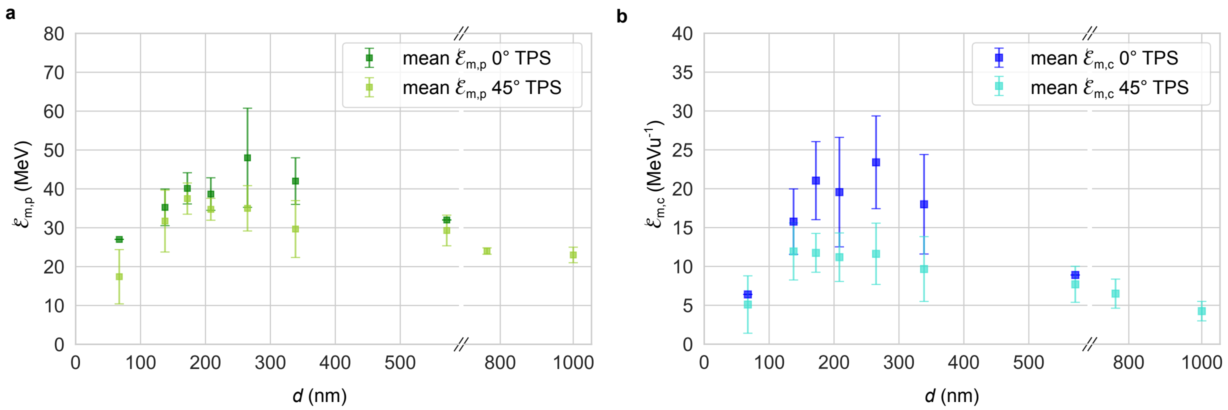

Figure S1: TPS data from J-KAREN-P experiment. Comparison of maximum ion energy $\mathcal{E}_m$ measured on the TPS at 0º and 45º for **a** protons and **b** C^{6+}/O^{8+} ions. The error bars represent the standard deviation of the experimental data in each target thickness bin.

Supplementary Figures 2 and 3: Temporal contrast comparison between J-KAREN-P and DRACO-PW

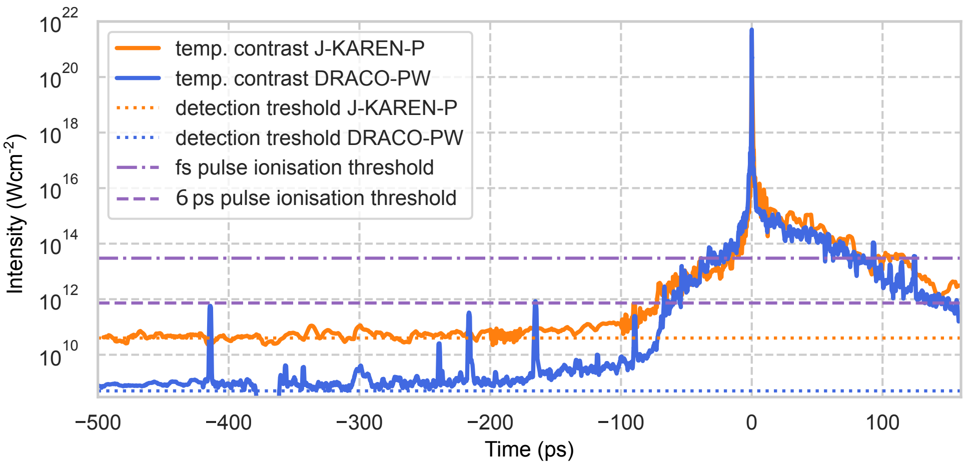

Figure S2: Comparison of temporal intensity contrast measurements. Experimental results from scanning third-order auto-correlator (Sequoia and SequoiaHD from Amplitude Technologies) measurements of J-KAREN-P (orange) and DRACO-PW (blue) laser pulse contrast up to 500 ps before the peak. The ASE level is less than 10^{11} and 10^{9} Wcm^{-2} respectively (noise level of detectors reached), and all visible short pre-pulses are below 10^{13} Wcm^{-2}.

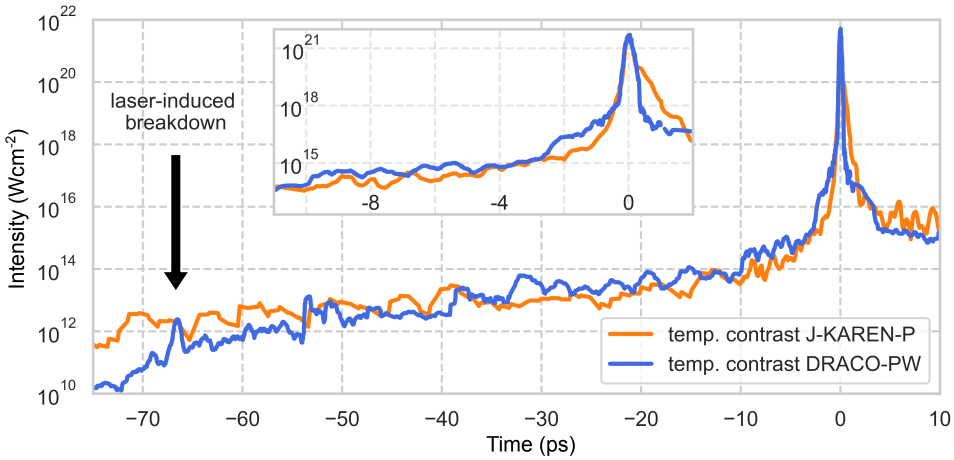

Figure S3: Comparison of temporal intensity contrast measurements. Experimental results from scanning third-order auto-correlator (Sequoia and SequoiaHD from Amplitude Technologies) measurements of J-KAREN-P (orange) and DRACO-PW (blue) laser pulse contrast up to 70 ps. The intensity during the rising edge of the coherent pedestal at −67 ps reaches the laser induced breakdown level. The similarity of the contrast levels between both laser systems implies a similar prepulse driven plasma expansion.

Supplementary Figure 4 and 5: plasma dynamics and ion acceleration for simulations without prepulse

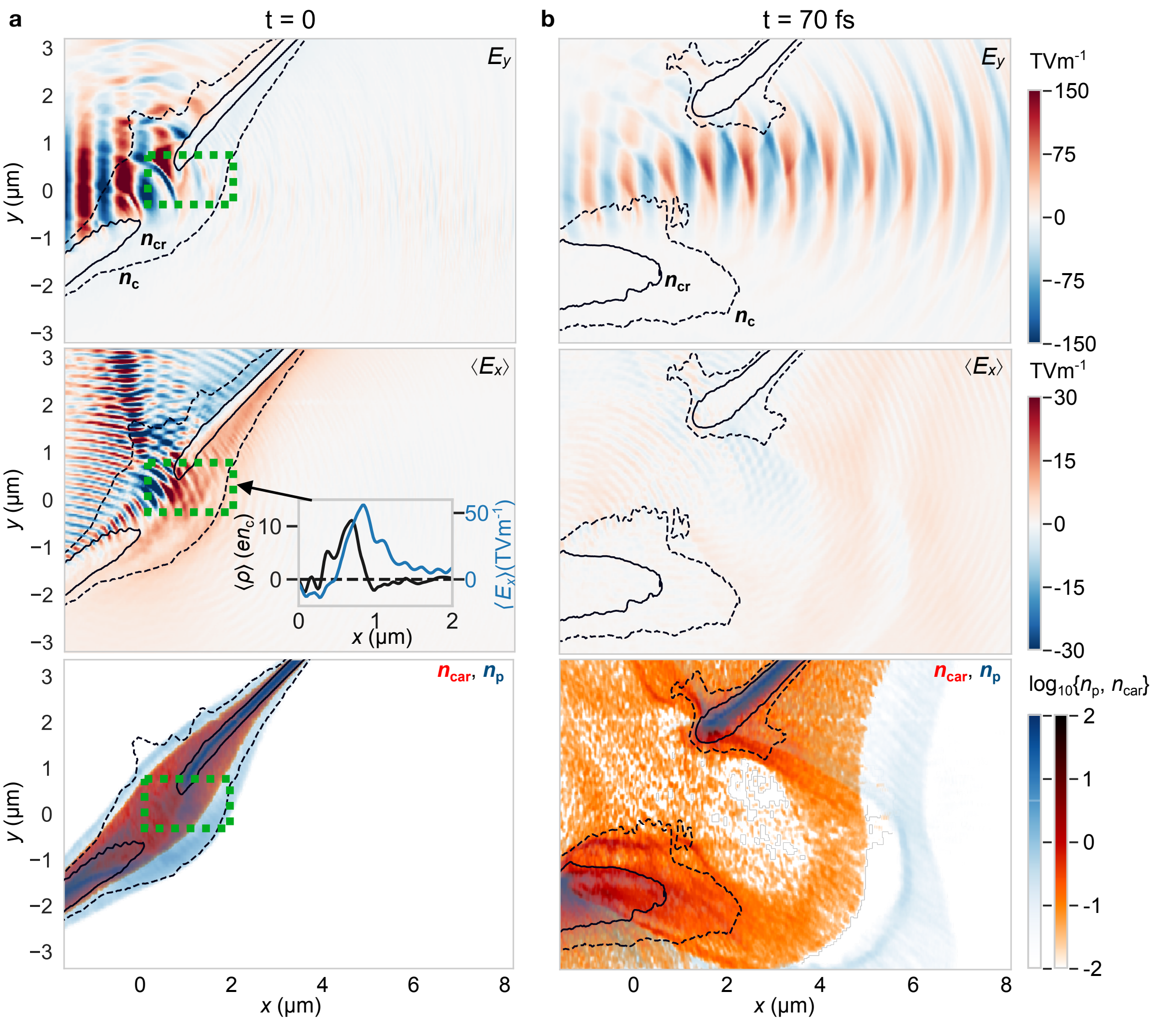

Figure S4: Dynamics of laser plasma interaction at optimum target thickness when not including prepulse into the simulation. **a** E_y, $\langle E_x \rangle$ and carbon/proton density (n_{car}, n_{p} normalised to n_{c}) for a 2D slice at $z = 0$ for a 65 nm target. The solid (dashed) contours give the relativistic (classical) critical electron density. Shown when the peak of the pulse arrives at the target, $t = 0$, for which the electron expulsion region is highlighted by the green box. The inset shows the cycle-averaged space charge density $\langle \rho \rangle$ and $\langle E_x \rangle$ for a lineout through the centre of the green box, averaging over $\Delta y = 400$ nm. **b** The same 2D slices shown at $t = 70$ fs, at the end of the main pulse interaction.

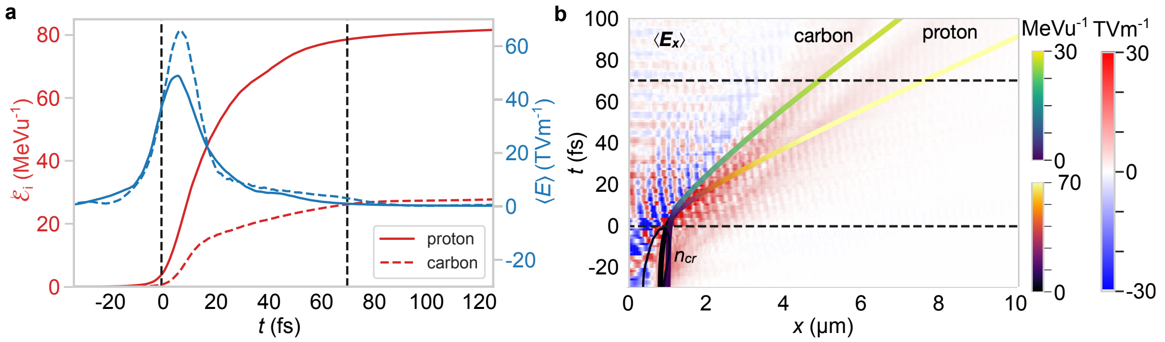

Figure S5: Particle tracking for simulations not including the prepulse. a Example of energy history (red) and local electric field (blue) $\langle E \rangle$ at the particle location for a typical energetic proton (solid lines) and carbon ion (dashed). **b** Time history of $\langle Ex \rangle$ in a lineout along x through the centre of the region of largest accelerating gradient, with the trajectory of the same proton and carbon ion overlaid. The black line shows the relativistically critical contour.
